# Temporal trend and spatial clustering of cholera epidemic in Kumasi-Ghana

**DOI:** 10.1038/s41598-018-36029-4

**Published:** 2018-12-14

**Authors:** Frank Badu Osei, Alfred Stein

**Affiliations:** Faculty of Geo-Information Science and Earth Observation (ITC), University of Twente, Enschede, Netherlands

## Abstract

Knowledge of the temporal trends and spatial patterns will have significant implications for effective preparedness in future epidemics. Our objective was to investigate the temporal trends and the nature of the spatial interaction of cholera incidences, dwelling on an outbreak in the Kumasi Metropolis, Ghana. We developed generalized nonparametric and segmented regression models to describe the epidemic curve. We used the pair correlation function to describe the nature of spatial clustering parameters such as the maximum scale of interaction and the scale of maximal interaction. The epidemic rose suddenly to a peak with 40% daily increments of incidences. The decay, however, was slower with 5% daily reductions. Spatial interaction occurred within 1 km radius. Maximal interaction occurred within 0.3 km, suggesting a household level of interactions. Significant clustering during the first week suggests secondary transmissions sparked the outbreak. The nonparametric and segmented regression models, together with the pair correlation function, contribute to understanding the transmission dynamics. The issue of underreporting remains a challenge we seek to address in future. These findings, however, will have innovative implications for developing preventive measures during future epidemics.

## Introduction

Cholera remains a public health threat in many developing countries despite remarkable research progress. Out of the 42 countries reporting cholera cases in 2014, 19 countries in Africa reported 55.25% of the cases and 84.36% of the deaths^1^. Ghana together with Nigeria, the Democratic Republic of Congo, Haiti, and Afghanistan recorded 84% of the 2014 cases worldwide^1^. In both endemic and non-endemic regions, effective cholera preparedness plans during outbreaks are based on lessons learned from previous outbreaks. Key questions include the nature of the epidemic curve, the speed of the spread, the transmission route, and clustering characteristics, i.e. scale of maximal clustering and maximal scale clustering. Studying the temporal trends and spatial heterogeneities can provide answers to these questions. This can also infer relevant environmental and climatic risk factors, examine etiological hypothesis and routes of transmission^2–5^.

Africa has largely reported the majority of cholera epidemics and deaths since the seventh pandemic established its new home on the continent^6^. A systematic review on the environmental determinants of cholera in Africa showed that inland (non-coastal) epidemics constituted a major part of the continental burden^7^. More than three-quarters of all cholera cases reported in sub-Saharan Africa in 2009–2011 affected inland regions. Thus, inland cholera is emerging as a relevant and major epidemiological concern in Africa. This stimulates efforts to understand the spatiotemporal heterogeneities of inland cholera epidemics. Yet the endemic and epidemic cholera dynamics have mostly been studied in coastal areas such as Bangladesh^8–10^, India^11^, Mexico^12^, Peru^13^, and some coastal African countries^14–18^ where there is close contact between infected populations and the estuarine (or riverine) environment.

In this study, we investigate the temporal trend and spatial clustering of cholera, drawing on the data collected during an epidemic in Kumasi, Ghana. Our objectives are to answer the following epidemiological questions: (1) what is the nature of the epidemic curve, and how different are the epidemic growth and decay gradients? (2) what is the maximum scale of spatial interaction and the scale of maximal interaction? We reason that answers to these questions will have important implications for effective cholera outbreak preparedness. We anticipate the spatial interaction to indicate higher than expected neighboring cases even at the beginning of the epidemic week^8^. The remainder of the manuscript is structured as follows; the next section describes the study area and the data. This is followed by a description of the statistical approaches, and then the results and analysis. The manuscript ends with some discussion and conclusions.

## Methods

### Study area and data

**Figure 1:**
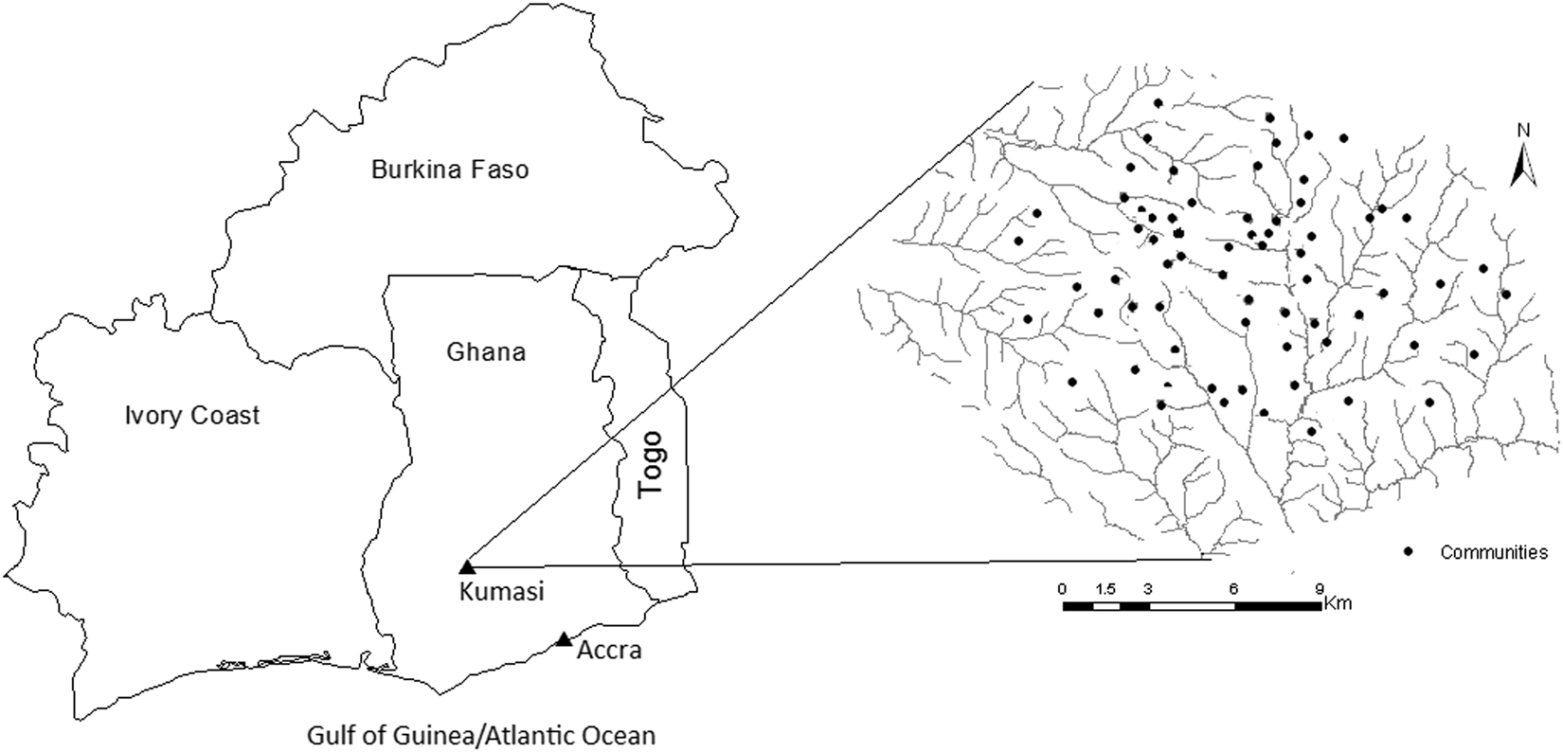
Map of Ghana and its neighbors (left), and Kumasi (right). This map was created using ArcGIS software (version 10.1, ESRI Inc. Redlands, CA, USA. https://www.esri.com/).

Kumasi is an inland city located in the south-central part of Ghana, 250 km northwest of Accra (Fig. 1). Ghana has been a known country of occasional cholera outbreaks since the 1970’s^19^. The recent outbreak in 2014 which recorded over 28,000 cases was mainly concentrated in coastal districts and their neighboring districts with only marginal cases in inland districts. Kumasi has occasionally been hit by a series of cholera outbreaks of which the 2005 outbreak has been the most severe. Hence our study focused on the 2005 outbreak. This outbreak started from the last week of September, lasting for a period of 72 days, which was the rainy season. The first confirmed case was recorded on 29th September 2005. In Ghana, it is mandatory for all reporting facilities (i.e. hospitals, clinics, and community volunteers) to report cholera cases to the Disease Control Unit (DCU). The DCU is purposely established to ensure effective surveillance of all communicable diseases (personal communication with the head of DCU, Ashanti region). A case definition of cholera was based on the WHO’s definition of clinical diagnosis which depends on whether or not the presence of cholera has been demonstrated in the area. The first case of cholera, however, was confirmed by bacteriological tests (personal communication with DCU director). In this study, only cholera cases made known to the Kumasi Metropolitan DCU through reporting facilities such as community volunteers, community clinics, and hospitals were used. The data obtained consisted of individual surveillance and laboratory records of cholera. The DCU registered cases with the following information: name, date of onset, date of reporting, gender, locality (community or suburb), sub-locality (description of residence), and age. For the preservation of confidentiality, we ensured the DCU officer in charge deleted the names of affected individuals before receipt of the data. The exact residential addresses of the cases were not recorded, however, the sub-locality field provided information regarding descriptions of their residencies. We determined the locations (mostly sub-localities) of the cases using a Global Positioning System (GPS). The geographic coordinates (latitudes and longitudes) in the WGS 84 datum were then transformed into the Ghana Transverse Mercator (GTM) coordinate system using a transformation program written by one of the authors (FBO). For cases that we could not trace their sub-localities in the database, we assigned the centroids of their localities of residence. Complete spatiotemporal information existed for the 1166 cholera cases we obtained from the DCU. Since the data were secondary, properly anonymized and informed consent was obtained by the DCU at the time of original data collection, ethical approval was not required.

### The empirical epidemic curve

To describe the nature of the epidemic curve, we developed a generalized nonparametric model for the daily incidences *chol*_*t*_, *t* = 1,…,.*T*^20,21^. We modeled *chol*_*t*_ as realizations from the Poisson distribution, *chol*_*t*_ ~ Poisson(*ϑ*_*t*_*µ*_*t*_), where *µ*_*t*_ is the mean and *ϑ*_*t*_ is a positive-valued random variable to account for over-dispersion, a situation where E(chol|t) < *V*(chol|t). Here, *ϑ*_*t*_ has mean equal 1 and variance *a* (the over-dispersion parameter) such that the marginal mean and variance are E(chol|t) = µ and *V*(chol|t) = µ + *a*µ^2^. Since the Gamma distribution is a conjugate of the Poisson distribution we chose *ϑ* ~ Gamma(1/*a*,1/*a*, one-parameter Gamma distribution with mean E(*ϑ*) = 1 and variance V(*ϑ*) = *a*. In so doing, the marginal distribution of *chol* is equivalent to the negative-binomial distribution *chol*_*t*_ ~ *NB*(*µ*_*t*_,*a*). We used the canonical log-link function to linearize the expected counts log(E[*chol*_*t*_]) = log(*µ*_*t*_) through the predictor log(*µ*_*t*_) β_0+*f*_ (*t*)., where *f*(t) is a nonlinear function of time *t* and *β*_*0*_ is the intercept. For the function *f*(*t*), we used penalized splines with truncated power basis functions $1,\,t,{t}^{2},\ldots ,{t}^{p}$, ${{(t-{\kappa }_{1})}^{p}}_{+}\,,\ldots ,\,{(t-{\kappa }_{M})}_{+}^{p}\,$ of degree *p*. Such basis functions with p ≥ 2 have continuous first derivatives without sharp corners resulting in an aesthetically appealing fit. This results in the *pth* degree spline model of the *m* = 1, …, *M* ascending clustered knots, ${\kappa }_{1},\ldots ,{\kappa }_{M}$, with *M* = max(*T*/4,20)^22^, that cover the ace of *t*. Thus,

1 $$\mathrm{log}({\mu }_{t})={\beta }_{0}+{\beta }_{1}{t}_{t}+{\beta }_{1}{t}_{t}^{2}+\cdots +{\beta }_{p}{t}_{t}^{p}+{\sum }_{m=1}^{M}{\beta }_{pm}{(t-{\kappa }_{m})}_{+}^{p}$$

where ${(t-{\kappa }_{k})}_{+}=(t-{\kappa }_{m})\times I(t > {\kappa }_{m})$ with $I(\cdot )$ the indicator function equals to one if *t* > κ and zero otherwise. With ${\bf{t}}=[1\,{t}_{t}^{1}{t}_{t}^{2},\ldots ,{t}_{t}^{p}]$ and ${\bf{Z}}=[{(t-{\kappa }_{1})}_{+}^{p},\ldots ,{(t-{\kappa }_{M})}_{+}^{p}]$. as vector-matrices, we reparametrized the model as a generalized mixed model representation $\mathrm{log}({\boldsymbol{\mu }})={\bf{t}}\hat{{\boldsymbol{\beta }}}+{\bf{Z}}\hat{{\bf{b}}}$, and estimated the parameters $\hat{{\boldsymbol{\beta }}}=\{{\beta }_{0},{\beta }_{1},{\beta }_{2},\ldots ,{\beta }_{p}\}$, $\hat{{\bf{b}}}=\{{\beta }_{p1},\ldots ,{\beta }_{pM}\}$ by maximizing the Penalized Quasi-likelihood^23^. Since the degree of the basis functions can influence the predictive performance, we fitted seven different models of varying degrees $p=1,\ldots ,6$. The same number of knots *M* were used for all the models. We used the chi-square goodness-of-fit (GOF) test on the null and model (residual) deviances to assess the adequacy of the models. We estimated the predictive performances using the generalized cross-validation (GCV) method

2 $$GCV(p)=\sum _{i=1}^{n}{(\frac{\{(I-{S}_{p})\mathrm{log}cho{l}_{t}\}i}{1-{n}^{-1}tr({S}_{p})})}^{2}$$

where *S*_*p*_. is a smoother matrix and dependent upon the degree *p* of the basis functions. The model that minimizes *GCV(p)* is the model with the best predictive performance. We used the R statistical software^24^ for this modeling.

### Epidemic growth and decay

We fitted a segmented Poisson regression model with an unknown single time break-point ${\kappa }_{peak}\,$to assess the difference in gradients between the epidemic growth and decay and to separate cases which occurred during the growth and decay periods for further analysis. The results of the models for the epidemic curve in the previous section give an indication that a sgle time break-point separates the epidemic growth and decay. Since this break-point is not known a priori, we expressed

3 $$\mathrm{log}({\mu }_{t})={\beta }_{0}+{\beta }_{1}{t}_{t}+{\beta }_{2}{({t}_{t}-{\kappa }_{peak})}_{+}$$

where the parameter *β*_1_ is the growth gradient and *β*_2_ is the difference in gradients between the growth and decay. This implies *β*_1_ + *β*_2_ is the decay gradient. We reparametrized the model as

4 $$\mathrm{log}({\mu }_{t})={\beta }_{0}+{\beta }_{1}{t}_{t}+{\beta }_{2}{({t}_{t}-{\kappa }_{peak})}_{+}+{\delta }_{\kappa }I{({t}_{t}-{\kappa }_{peak})}^{-}$$

and estimated the parameters iteratively until the indicator function $\,I{()}^{-}$ converged to zero. Thus a generalized linear model is fitted at each iteration and the break-point value is updated via ${\hat{\kappa }}_{peak}={\hat{\kappa }}_{peak}+{\hat{\delta }}_{\kappa }/{\hat{\beta }}_{2}$, where ${\hat{\delta }}_{\kappa }$ measures the estimated gap between the two fitted lines^25,26^. Convergence is achieved when the break-point gap ${\hat{\delta }}_{\kappa }\,$becomes close to zero; here, ${\hat{\kappa }}_{peak}\,$is the optimal time at which the epidemic reaches a peak. In order to check the adequacy of the model fit, we employed the Davies test of hypothesis^25,27^ on the break-point to determine if the difference in slopes ${\hat{\beta }}_{2}$ is significantly different from zero. We further used the estimated break-point time ${\hat{\kappa }}_{peak}$ to dichotomize cases into growth and decay. We used the *Segmented*^25^ package of the R statistical software^24^ for this modeling. We used the chi-square GOF test on the null and model deviances to assess the model adequacy.

### Spatial interactions

To estimate the maximum scale of interaction and the scale of maximal interaction, we used the pair correlation function $g(r)$. If the occurrences of cholera cases interact, the number of cases around any chosen case within a radius $r$ will be more than expected, an exhibition of spatial interaction. In order to test this, we considered the occurrences of cholera cases $cho{l}_{i}=({x}_{1i},{x}_{2i})$ as realizations of a spatial point process within the window ${\boldsymbol{W}}$, where $({x}_{1i},{x}_{2i})$ are the geographic coordinates. Then for the infinitesimal regions ${\rm{\Delta }}v$ and ${\rm{\Delta }}u$ centered on locations $v$ and $u$ within ${\boldsymbol{W}}$, the theoretical pair correlation function is $\,g(r)={\rho }_{(v,u)}/{\lambda }_{v}{\lambda }_{u}$, where ${\lambda }_{v}$, ${\lambda }_{u}$ and ${\rho }_{(v,u)}\,$are the first and second order intensities, respectively. Here, $|{\rm{\Delta }}v|\,$and $|{\rm{\Delta }}u|$ are the areas of the regions ${\rm{\Delta }}v$ and ${\rm{\Delta }}u$, and $N({\rm{\Delta }}v)$ and $N({\rm{\Delta }}v)$ are the numbers of cholera cases within the regions, respectively. The first order intensity describes the spatial inhomogeneity whereas the second order intensity describes the spatial interaction between cases. We estimated the empirical pair correlation function $\hat{g}(r,\,\sigma )\,$using

5 $$\hat{g}(r,\,\sigma )=\frac{1}{2\pi }\sum _{u=1}\sum _{v\ne u=1}\frac{{\gamma }_{\sigma }(r-\parallel u-v\parallel )}{{e}_{uv}u\parallel u-v\parallel {\hat{\lambda }}_{v}{\hat{\lambda }}_{u}}$$

where ${e}_{uv}$ is an edge correction weight, ${\gamma }_{\sigma }\,$is a one-dimensional kernel function with smoothing bandwidth $\sigma  > 0$, ${\hat{\lambda }}_{v}$ and ${\hat{\lambda }}_{u}\,$are estimated intensities at locations *v* and *u* and depend on the Euclidean distance $\,r=u-v$ between occurrences *chol*_*u*_ and *chol*_*v*_. We chose Ripley’s isotropic edge correction which expresses *e*_*uv*_ as the fraction of the length of the circle of radius $u-v$ lying within ${\boldsymbol{W}}$. Thus if the circle centered on *i* with radius $u-v$ is completely within the study plot, ${e}_{uv}=1$; otherwise, it is the proportion of that circle’s circumference within the plot^28^. The *Epanechnikov kernel* provides asymptotically optimal convergence rate for both the mean integrated square error and the mean square error, hence it is used in this study. Although other alternatives like truncated quadratic functions exist, the optimality of the *Epanechnikov kernel* has established it as the usual choice in point pattern analysis^29^. We used the denominator $u-v$ instead of the usual radii *r* because for small radii *r*, the variance of $\hat{g}(r,\,\sigma )\,$becomes infinity. It is conceivable that the first-order intensities ${\hat{\lambda }}_{v}$ and ${\hat{\lambda }}_{u}$ are affected by location-specific environmental conditions, hence they were estimated as a function of location. Thus, we estimated the intensity at any location *u* of neighboring cases *chol*_*i*_, *i* = 1, …, *m* within the bandwidth *σ* as ${\hat{\lambda }}_{u}={\sum }_{i=1}^{m}{e}_{iu}^{-1}{\gamma }_{\sigma }(u-{x}_{i})$^29^. Under the null hypothesis of no interaction between cholera cases, $\hat{g}(r,\,\sigma )=1$; whereas $\hat{g}(r,\,\sigma ) > 1$ indicates interaction and $\hat{g}(r,\,\sigma ) < 1$ indicates inhibition or repulsion between cases. We used the *spatstat* package^29^ of the R statistical software^24^ for estimating $\hat{g}(r,\,\sigma )$.

## Results and Analysis

### The empirical epidemic curve

**Figure 2:**
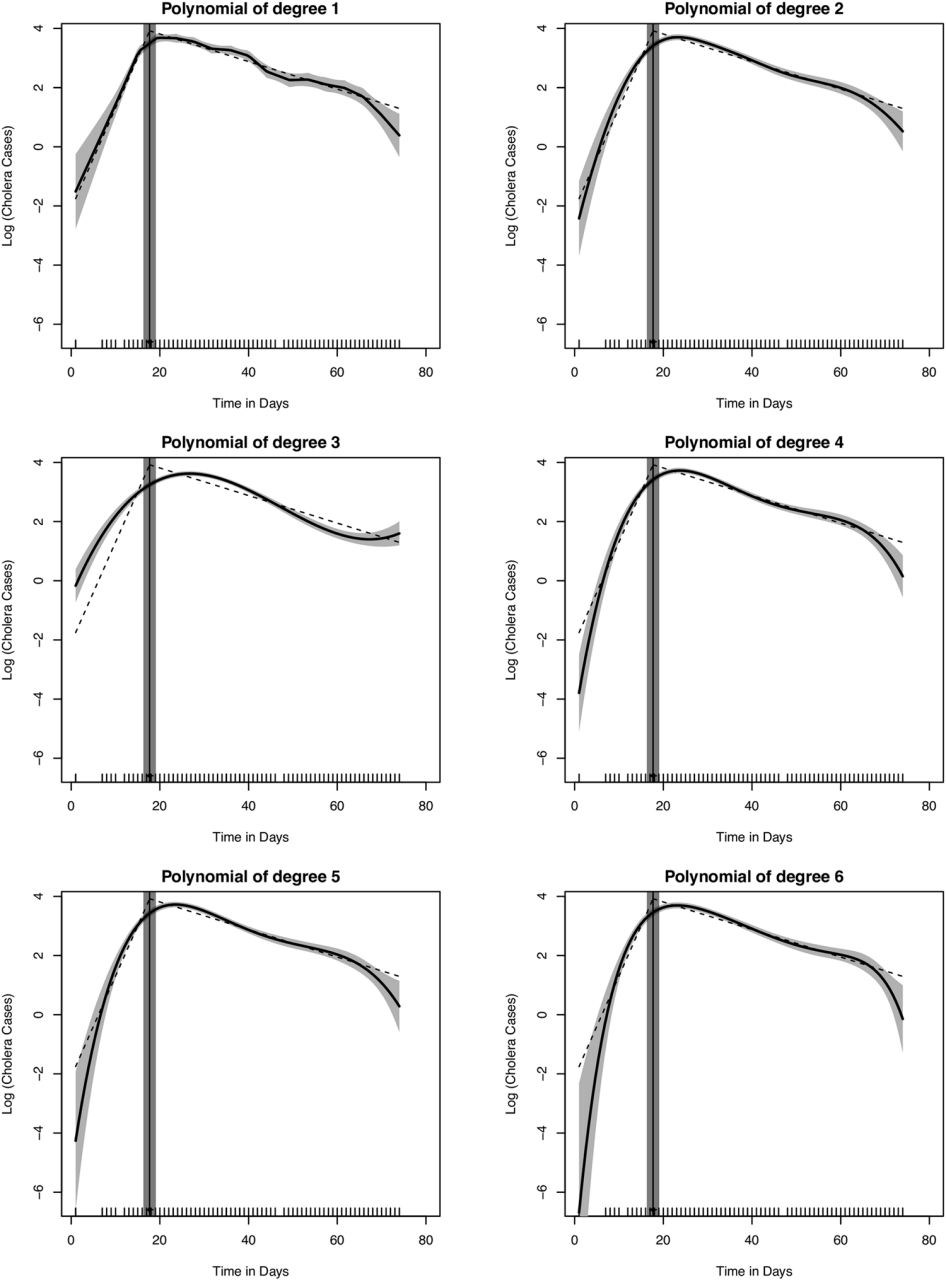
Epidemic curves of the generalized nonlinear models for *p* = 1,…,7 (solid lines) and the segmented regression (dashed lines). This graph was created using the R statistical software (version 3.4.2, https://cran.r-project.org).

The plot of the empirical epidemic curves and the standard error bands are shown in Fig. 2. We fitted seven different models for the epidemic curve for $\,p=1,\ldots ,6$. We obtained adequate fit at 5% significance level for all models under the chi-square GOF test as the residual deviances were less than the upper chi-square critical values (Table 1).

**Table 1:** Parameters of the chi-square GOF test for model adequacy assessment.

Model	DF	Null Deviance	Residual Deviance	5% Critical value
1	53	466.85	63.15	70.99
2	52	508.94	61.00	69.83
3	51	501.02	61.31	68.67
4	50	507.82	60.63	67.51
5	49	508.97	61.01	66.34
6	48	515.31	61.02	65.17

**Figure 3:**
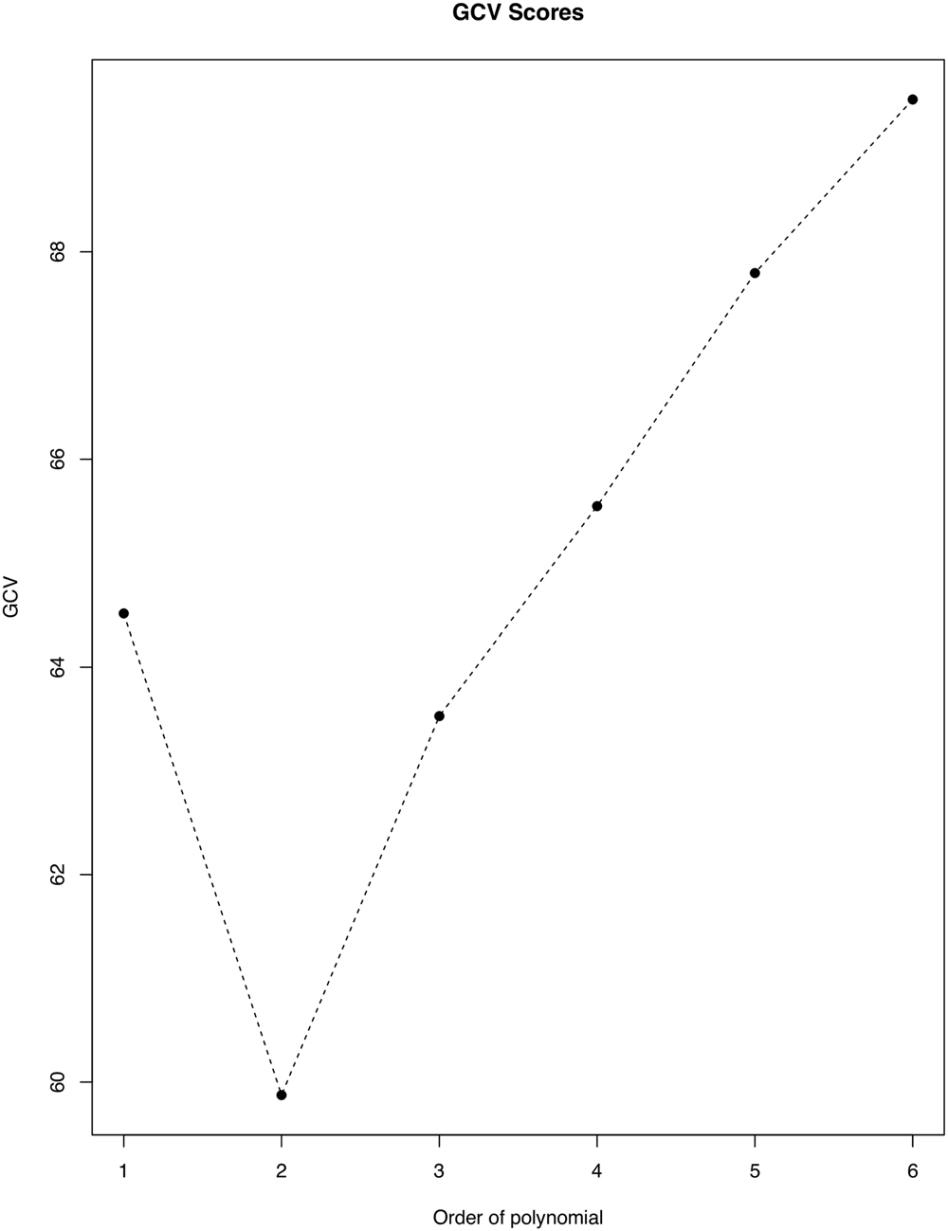
Generalized cross-validation curve for model predictive performances. Lower cross-validation value implies better predictive performance. This graph was created using the R statistical software (version 3.4.2, https://cran.r-project.org).

We also compared the predictive performances of the models using *GCV(p)*. Although higher order polynomials are expected to produce smoother curves, we observed systematic decreases in the prediction performance with increasing *p* for Model 2 (*p* = 2) to Model 6 (*p* = 6). The linear polynomial model, Model 1 (*p* = 1), has larger *GCV(p)* than Models 2 and 3 and thus has a greater bias because it is continuous, but not differentiable at the knots. Although this model is under-smoothed and has a less aesthetic appearance, its estimates are unexpectedly superior to those with *p* > 3. Model 2 (*p* = 2), the quadratic polynomial model, has the highest predictive performance as its smoothing matrix comparatively minimizes *GCV(p)* (Fig. 3). All the modeled curves are comparable in shape, except Model 3 which appears to remain constant with time towards the end. Dwelling on Model 2, the resulting prediction model equation for the epidemic curve is $\mathrm{log}({\mu }_{t})={\beta }_{0}+{\beta }_{1}{t}_{t}+{\beta }_{2}{t}_{t}^{2}+{\sum }_{m=1}^{2}{\beta }_{3m}{(t-{\kappa }_{m})}_{+}^{2}$.

### Epidemic growth and decay

The null and model deviances for the segmented regression model were 356.5 and 69.7 under 68 and 65 degrees of freedom (DF), respectively. Under the null hypothesis that our model is correctly specified, the model deviance of 69.7 falls below the critical region at 5% significance level on 65 DF of the chi-square distribution. Hence, we conclude that our model adequately fits the data based on the chi-square GOF test. This model indicates that the difference in gradients between the growth and decay (${\beta }_{2}=-\,0.387$, *p value* < 0.001) is significantly different from zero (Table 2). The estimated break-point time was $\,{\hat{\kappa }}_{peak}=17.7$ (*standard error* = 0.374) and approximated to the 18^th^ day. The growth of the epidemic was characterized by a significant increasing gradient of ${\beta }_{1}=0.340$ on the log scale (*p value* < 0.001), indicating a multiplicative effect of ${e}^{{\beta }_{1}}=1.40$ or 40% increments in the daily number of new cases (Fig. 2). The decay of the epidemic was characterized by a significant decreasing gradient of ${\beta }_{1}+{\beta }_{2}=-\,0.047$ (*p value* < 0.001) on the log scale, indicating a less rapid decline with a multiplicative effect of ${e}^{{\beta }_{1}+{\beta }_{2}}=0.95$ or 5% reductions in the daily number of new cases.

**Table 2:** Parameter estimates of the segmented Poisson regression model.

Parameter	Estimate	Standard Error
${\beta }_{0}$	−2.101	0.569
${\beta }_{1}$	0.340	0.038
${\beta }_{2}$	−0.387	0.038
${\hat{\kappa }}_{peak}$	17.7	0.374

### Spatial interactions

**Figure 4:**
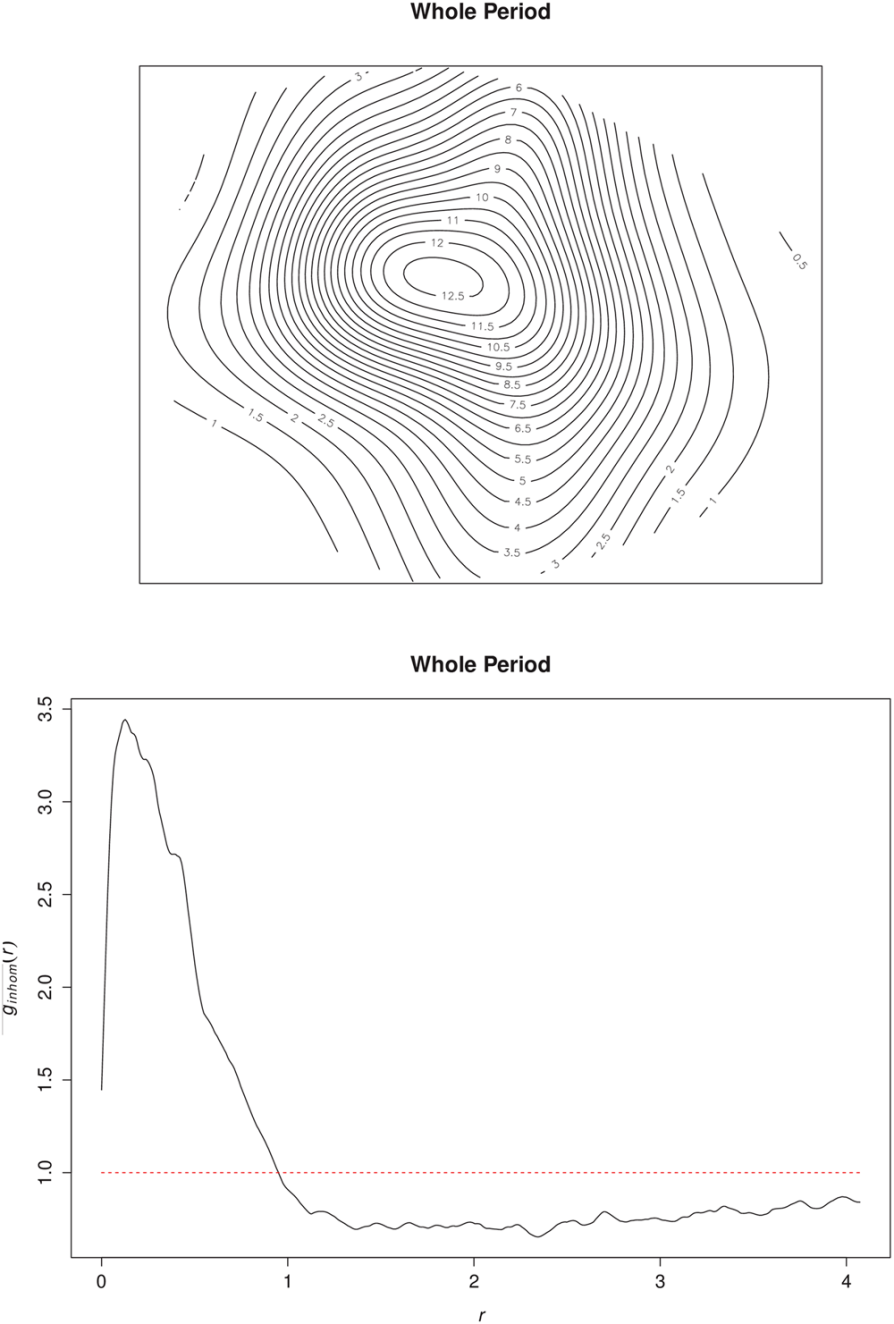
Spatial intensities and pair correlation curve for the whole epidemic period. Spatial distribution of the heterogeneous intensities for the whole epidemic period (Top) and the corresponding pair correlation curve (Bottom). The dashed line represents the line of complete spatial randomness. These graphs were created using the R statistical software (version 3.4.2, https://cran.r-project.org).

**Figure 5:**
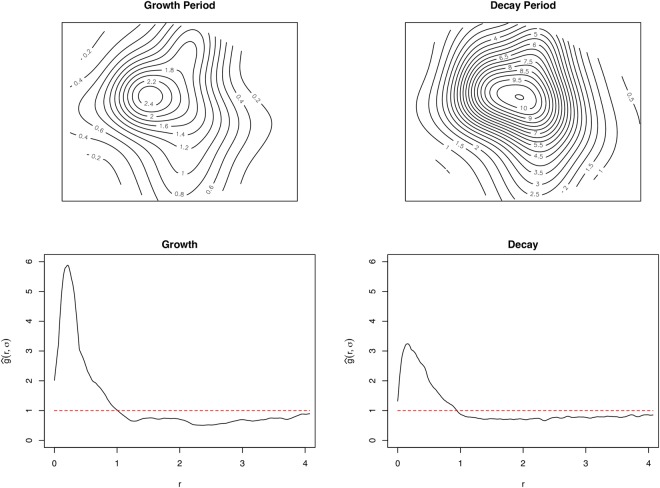
Spatial intensities and pair correlation curves for epidemic growth, decay. Spatial distribution of the heterogeneous intensities for the epidemic growth, decay (Top) and their corresponding pair correlation curves (Bottom). The dashed lines represent the line of complete spatial randomness. These graphs were created using the R statistical software (version 3.4.2, https://cran.r-project.org).

**Figure 6:**
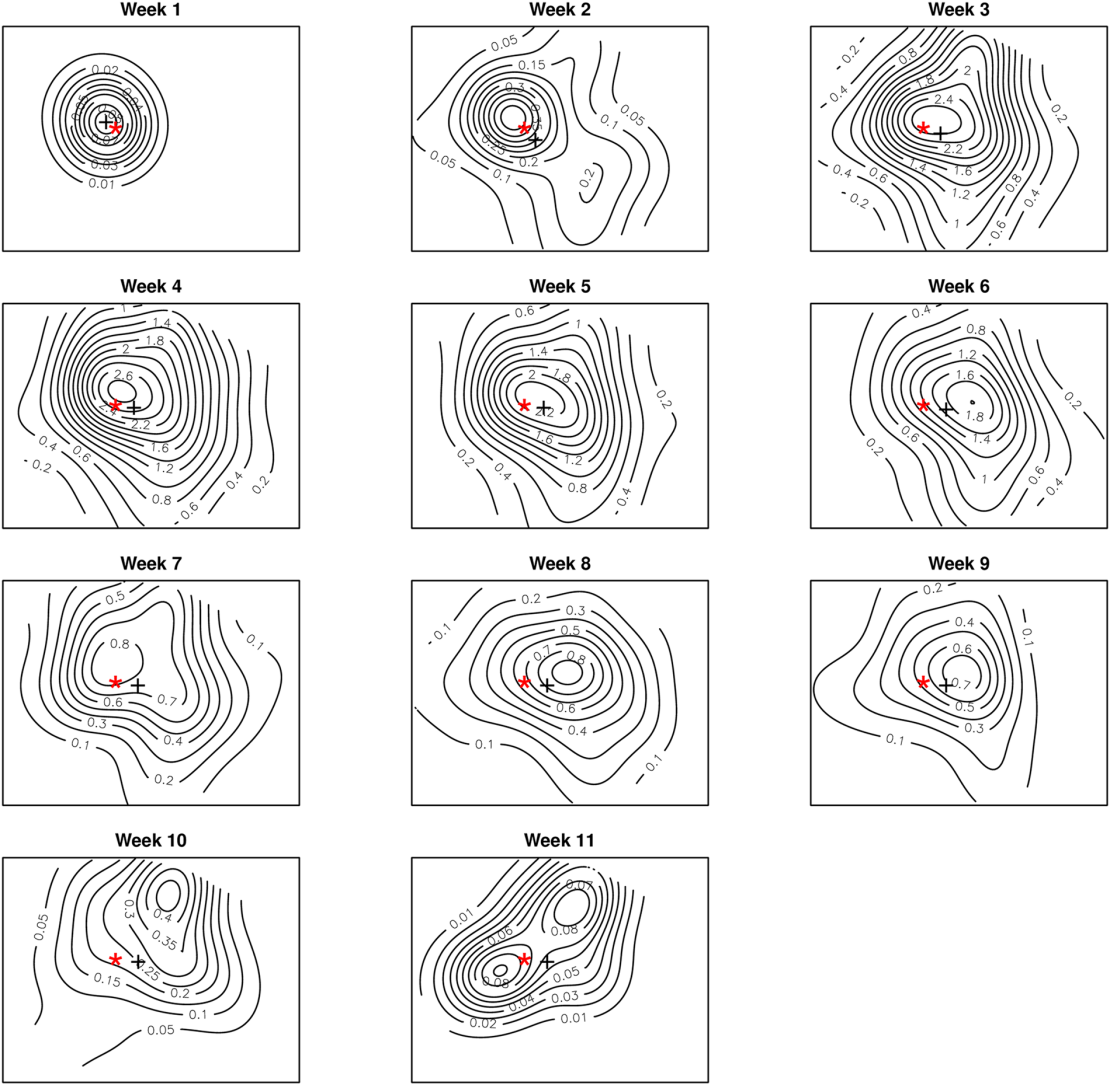
Spatial distribution of the heterogeneous intensities for weeks 1 to 11. The symbol “*” indicates the location of the index case and “+” indicates the epicenter. These were created using the R software statistical software (version 3.4.2, https://cran.r-project.org).

The estimated average intensity was 4.4 cases km^−2^. The intensity of cases varied heterogeneously across the study window with an outward decreasing trend from the central part, ranging from ≈ 0.5 cases per *km*^2^ within the peripheries to ≈13 cases per *km*^2^ towards the central parts (Fig. 4). Varied intensities were observed between the weekly incidences (Figs 5 and 6). The weekly occurrences also showed high intensities within the central parts with systematic reductions towards the peripheries (Fig. 6). The epidemic center (center of mass of the locations of cases) remained virtually the same and deviated only marginally from the index case location during the growth period, indicating stationarity. We observed similar outward decreasing trend patterns of spatial intensities for the growth and the decay periods (Figs 5 and 6).

**Figure 7:**
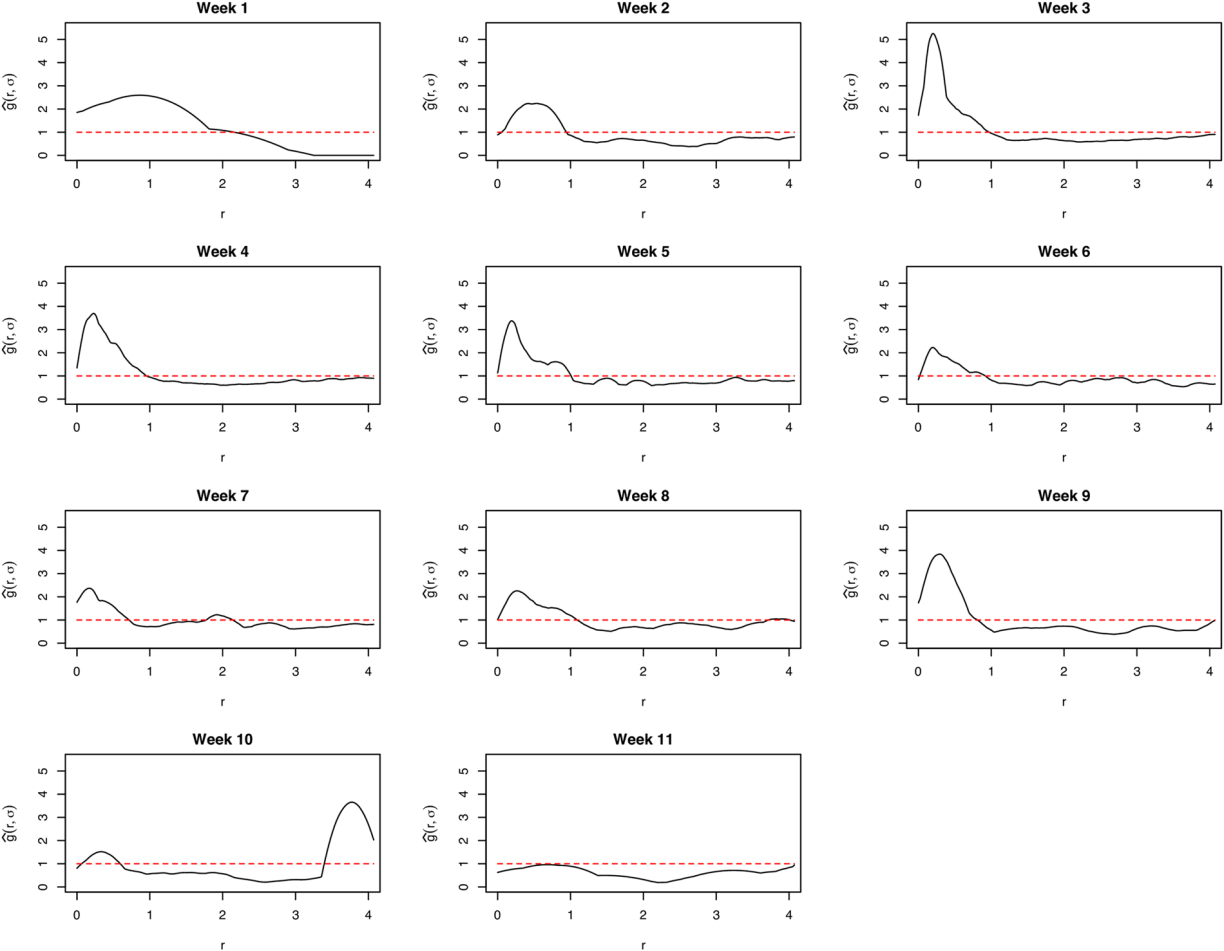
Pair correlation curves for weeks 1 to 11. The dashed lines represent the line of complete spatial randomness. These were created using the R statistical software (version 3.4.2, https://cran.r-project.org).

The $\hat{g}(r,\,\sigma )$ curves for the whole epidemic period showed the existence of an interaction between cholera cases within 1 km range, as the curve is well above the line of complete spatial randomness at this distance (Fig. 4). For distances greater than 1 km, the interaction decreases to repulsion or inhibition as the curve falls below the line of complete spatial randomness. We observed a short-range distance of maximal interaction as low as ≈0.3 km (Fig. 4). Significant levels of interaction were within ≈1 km range for the first week of the epidemic (week 1) through to week 10 as the $\hat{g}(r,\,\sigma )$ curves were all well above the line of complete randomness (Fig. 7). Similar maximal distances of interaction and distances of maximal interaction were observed to be ≈1 km and ≈0.3 km, respectively, regardless of the specific week of the epidemic, except the last week. The levels of interaction, however, differed with the highest observed in the third week (Fig. 7). The observed levels of interaction for the growth period were particularly higher than those for the decay period as well as for the entire epidemic period (Fig. 4).

## Discussion

The nonparametric model for the daily counts illuminated the nature of the epidemic curve, whereas the segmented regression model showed the dissimilarities between the growth and decay gradients. The epidemic was characterized by a rapid rise to a peak and then a slower decline in the number of occurrences. The growth and decay gradients were markedly different with a relatively prolonged and reduced rate of the decay. The shortened length of the growth at just the 18^th^ day of the epidemic can be attributed to early public health interventions after notice and declaration of the cholera outbreak. As early as the second week of the epidemic, there were various health promotion campaigns and education on both radio, television and print media by the DCU. Such early response is characteristic of the DCU of Ghana. Though, if it were complemented with improved hygiene by households, and improved water and sanitation by the government, the cases could have been reduced to the barest minimum.

The epidemiological implication of the rapid growth to the peak may include lack of awareness and knowledge of the disease when *V. cholerae* is suddenly present in the population. Thus, during the growth period, fewer people in the population are aware of an outbreak; hence, precautionary measures would be less, leading to high susceptibility. The rapid growth could have also been induced by the dominant role of secondary cholera transmissions due to the hyper-infective nature of the bacteria after excretion from infected people. Studies have suggested differences in the genes of *V. cholerae* responsible for primary transmissions (i.e., those from the aquatic environment) and those responsible for secondary transmissions (i.e. those excreted from infected individuals). When inoculated into the intestines of mice via gavage feeding, freshly shed *V. cholerae* greatly out-competes bacteria grown *in vitro*, by as much as 700-fold^30–32^. The cholera bacteria become hyper-infective when excreted from infected persons and are thought to be responsible for faster bacterial growth in the gastrointestinal tract and increased shedding. In a mathematical model to understand the transmission dynamics, Mukandavire *et al*.^33^ also found that secondary transmissions contributed to the sustenance of cholera in Zimbabwe, a landlocked country.

Estimates of the maximum scale of interaction, the scale of maximal interaction, and the week the interaction began convey information that reflects the transmission mechanisms during the outbreak. Such information indicates the prospects for effective control of future cholera outbreaks and for designing targeted surveillance programs. Although the interaction of incidences was significant within ≈1 km range, its dominance within ≈0.3 km suggests high occurrences of short distance transmission mechanism. This implicates household-level characteristics and contacts as enhancing one’s vulnerability to infection, suggesting the dominant role of secondary transmissions similar to that seen in earlier studies elsewhere^34,35^. A probable explanation regarding short distance transmission mechanisms is the prevalence of domestic/household water storage due to intermittent water supply in the Kumasi Metropolis. Unhygienic handling practices of stored water by households can exacerbate cholera infection, as suggested to be the case in other African countries like Malawi^36^, Guinea-Bissau^37,38^, and Kenya^39–41^.

Significant spatial interaction occurred at the beginning (within the first week) of the epidemic, signifying the responsible role of secondary transmission in sparking the outbreak. This explanation is deduced from Miller at al^42^ who suggested that no spatial interaction should exist between cases when primary transmission sparks the epidemic. This aspect of secondary transmission sparking the epidemic arguably suggests the absence of primary transmission during the outbreak. This diverges from the roles of primary and secondary transmissions observed in coastal endemic regions^8,32,42,43^. Additional support for this argument derives from the stationarity of the epidemic center during the growth period, reflecting that cholera widely spread from its initial source of contamination until its peak^8^. Based on our argument that secondary transmission sparked the outbreak, one could only suggest that the initial cholera case must have been imported from elsewhere, probably near the coastal regions of Ghana. A rebuttal, however, should be supported by successful isolation of *V. cholerae* in our study area during inter-epidemic periods. However, in inland regions where cholera outbreaks are sporadic, the environmental reservoir where the cholera *vibrios* retreat to after epidemics remains elusive. In fact, isolation of the cholera *vibrios* in several inland areas have scarcely been successful during inter-epidemic periods^44,45^. During an epidemic period in Tanzania, *V. cholerae* was isolated from water samples from the Lumemo River, but not from any other water source^46^, indicating difficulties of the bacterial establishing natural environmental reservoirs in inland regions. That said, this finding diverges from the initial interpretation of cholera dynamics in coastal endemic regions by other authors where initial cholera cases are expected to be random without any apparent connection^8,42^. For instance, in coastal endemic regions like the Matlab area of Bangladesh, a hypothesized dispersal pattern of primary transmissions during epidemics has been established^8^. There, primary transmission sparks the outbreak at several distance locations whereby secondary transmission follows and dominates with the clustering of cases at relatively small scales. Besides, the isolation of both toxigenic and viable but nonculturable *vibrios* during inter-epidemic periods has widely been successful in such environments^47–50^.

Notwithstanding the significance of this study, some limitations should be mentioned. First, underreporting of cholera cases is a potential limitation. The failure of asymptomatic carriers or infections with mild symptoms to seek medical attention could lead to lower than actual cases of cholera. Due to a large number of cases reporting to the health facilities, not all cases could be biologically confirmed before treatment, and this could also lead to over-reporting of cases. Possible inherent data quality issues with respect to the accuracy of the GPS coordinates have not been considered in the study.

## Conclusions

This study presented a generalized nonlinear model for the epidemic curve of cholera, characterized the epidemic growth and decay gradients, and estimated the levels of spatial interaction at varying distance scales. The study provides intuitive inferences as we appropriately captured the underlying empirical structure of cholera. It can be extended to include both fixed and time-varying covariates. The pair correlation function was favorable to address the heterogeneous intensities and the clustering characteristics such as the maximal distance of interaction and the distance of maximal interaction. We found evidence of secondary transmissions in initiating the epidemic. Interactions between cases were within 1 km scale and were dominated within households. Thus, strengthening household level sanitation practices is critical in reducing infections from infected people. An additional conclusion regarding the consequences of the role of secondary transmission relates to strengthening surveillance to restrict cholera importation by infected individuals. Correcting for underreporting of cholera cases in studies like this remains an area of ongoing research which we seek to investigate in future^51^. Improvements in public health surveillance are key to reducing underreporting. To summarize, this study has shown the usefulness of generalized nonparametric and segmented regression models, and of the pair correlation function in extracting key epidemiological information. The results of this study may have important implications for public health decision making for developing effective cholera outbreak preparedness strategies.

## Data Availability

Available Upon Request.

## References

[1] W.H.O. *Weekly epidemiological record 90(40)*. (2015).

[2] Homan, T. *et al*. Spatially variable risk factors for malaria in a geographically heterogeneous landscape, western Kenya: an explorative study. *Malar. J*. **15**, (2016). 10.1186/s12936-015-1044-1 PMC4700570 26729363

[3] Rulisa S (2013). Malaria Prevalence, Spatial Clustering and Risk Factors in a Low Endemic Area of Eastern Rwanda: A Cross Sectional Study. PLOS ONE.

[4] Sluydts V (2014). Spatial clustering and risk factors of malaria infections in Ratanakiri Province, Cambodia. Malar. J..

[5] Szonyi B, Srinath I, Esteve-Gassent M, Lupiani B, Ivanek R (2015). Exploratory spatial analysis of Lyme disease in Texas -what can we learn from the reported cases?. BMC Public Health.

[6] Gaffga NH, Tauxe RV, Mintz ED (2007). Cholera: a new homeland in Africa?. Am. J. Trop. Med. Hyg..

[7] Rebaudet S, Sudre B, Faucher B, Piarroux R (2013). Cholera in coastal Africa: a systematic review of its heterogeneous environmental determinants. J. Infect. Dis..

[8] Ruiz-Moreno D, Pascual M, Emch M, Yunus M (2010). Spatial clustering in the spatio-temporal dynamics of endemic cholera. BMC Infect. Dis..

[9] Ali M, Emch M, Donnay JP, Yunus M, Sack RB (2002). Identifying environmental risk factors for endemic cholera: a raster GIS approach. Health Place.

[10] Ali, M., Emch, M., Donnay, J. P., Yunus, M. & Sack, R. B. The spatial epidemiology of cholera in an endemic area of Bangladesh. *Soc Sci Med* **55**, (2002). 10.1016/s0277-9536(01)00230-1 12220086

[11] Kanungo S (2010). Cholera in India: an analysis of reports, 1997–2006. Bull. World Health Organ..

[12] Borroto RJ, Martinez-Piedra R (2000). Geographical patterns of cholera in Mexico..

[13] Gil AI (2004). Occurrence and distribution of Vibrio cholerae in the coastal environment of Peru. Environ. Microbiol..

[14] Constantin de Magny G, Guégan J-F, Petit M, Cazelles B (2007). Regional-scale climate-variability synchrony of cholera epidemics in West Africa. BMC Infect. Dis..

[15] Fleming G, Merwe MVD, McFerren G (2007). Fuzzy expert systems and GIS for cholera health risk prediction in southernAfrica. Environ. Model. Softw..

[16] Luque Fernández MÁ (2009). Influence of temperature and rainfall on the evolution of cholera epidemics in Lusaka, Zambia, 2003–2006: analysis of a time series. Trans. R. Soc. Trop. Med. Hyg..

[17] Mendelsohn J, Dawson T (2008). Climate and cholera in KwaZulu-Natal, South Africa: The role of environmental factors and implications for epidemic preparedness. Int. J. Hyg. Environ. Health.

[18] Paz S (2009). Impact of temperature variability on cholera incidence in southeastern Africa, 1971–2006. EcoHealth.

[19] Pobee, J. O. M. & Grant, F. Case Report of Cholera. *Ghana Med. J*. 306–309 (1970).

[20] Karcher P, Wang Y (2001). Generalized Nonparametric Mixed Effects Models. J. Comput. Graph. Stat..

[21] Ruppert, D., Wand, M. & Carroll, R. *Semiparametric Regression*. (Cambridge University Press, Cambridge, 2003).

[22] Kaufman, L. & Rousseeuw, P. J. *Finding Groups in Data: An Introduction to Cluster Analysis*. (John Wiley & Sons, 2009).

[23] Breslow NE, Clayton DG (1993). Approximate Inference in Generalized Linear Mixed Models. J. Am. Stat. Assoc..

[24] R Core Team. R: A language and environment for statistical computing. R Foundation for Statistical Computing. (2016).

[25] Muggeo VM (2008). Segmented: an R package to fit regression models with broken-line relationships. R News.

[26] Muggeo VMR (2003). Estimating regression models with unknown break-points. Stat. Med..

[27] Davies RB (1987). Hypothesis Testing when a Nuisance Parameter is Present Only Under the Alternatives. Biometrika.

[28] Diggle, P. *Statistical Analysis of Spatial Point Patterns*. (Arnold, 2003).

[29] Baddeley, A., Rubak, E. & Turner, R. *Spatial Point Patterns: Methodology and Applications with R*. (CRC Press, 2015).

[30] Alam A (2005). Hyperinfectivity of human-passaged Vibrio cholerae can be modeled by growth in the infant mouse. Infect. Immun..

[31] Merrell DS (2002). Host-induced epidemic spread of the cholera bacterium. Nature.

[32] Hartley DM, Morris JG, Smith DL (2005). Hyperinfectivity: A Critical Element in the Ability of V. cholerae to Cause Epidemics?. PLoS Med..

[33] Mukandavire Z (2011). Estimating the reproductive numbers for the 2008–2009 cholera outbreaks in Zimbabwe. Proc. Natl. Acad. Sci. USA.

[34] Debes AK, Ali M, Azman AS, Yunus M, Sack DA (2016). Cholera cases cluster in time and space in Matlab, Bangladesh: implications for targeted preventive interventions. Int. J. Epidemiol..

[35] Weil AA (2009). Clinical Outcomes in Household Contacts of Patients with Cholera in Bangladesh. Clin. Infect. Dis. Off. Publ. Infect. Dis. Soc. Am..

[36] Swerdlow, D. L. *et al*. Epidemic cholera among refugees in Malawi, Africa: treatment and transmission. **118**, 207–214 (1997). 10.1017/s0950268896007352 PMC2808810 9207730

[37] Rodrigues A (2000). Protection from cholera by adding lime juice to food – results from community and laboratory studies in Guinea‐Bissau, West Africa. Trop. Med. Int. Health.

[38] Rodrigues A, Brun H, Sandstrom A (1997). Risk factors for cholera infection in the initial phase of an epidemic in Guinea-Bissau: protection by lime juice. Am. J. Trop. Med. Hyg..

[39] Mahamud AS (2012). Epidemic cholera in Kakuma Refugee Camp, Kenya, 2009: the importance of sanitation and soap. J. Infect. Dev. Ctries..

[40] Mugoya I (2008). Rapid spread of Vibrio cholerae O1 throughout Kenya, 2005. Am. J. Trop. Med. Hyg..

[41] Shultz A (2009). Cholera outbreak in Kenyan refugee camp: risk factors for illness and importance of sanitation. Am. J. Trop. Med. Hyg..

[42] Miller CJ, Feachem RG, Drasar BS (1985). In *Cholera epidemiology in developed and developing countries: new thoughts on transmission, seasonality, and control*. Lancet.

[43] Ruiz-Moreno D, Pascual M, Bouma M, Dobson A, Cash B (2007). Cholera Seasonality in Madras (1901–1940): Dual Role for Rainfall in Endemic and Epidemic Regions. EcoHealth.

[44] Birmingham ME (1997). In *Epidemic cholera in Burundi: patterns of transmission in the Great Rift Valley lake region*. Lancet.

[45] Tauxe RV, Holmberg SD, Dodin A, Wells JV, Blake PA (1988). Epidemic cholera in Mali: high mortality and multiple routes of transmission in a famine area. Epidemiol. Infect..

[46] Acosta CJ (2001). Cholera outbreak in southern Tanzania: risk factors and patterns of transmission. Emerg. Infect. Dis..

[47] Binsztein N (2004). Viable but Nonculturable Vibrio cholerae O1 in the Aquatic Environment of Argentina. Appl. Environ. Microbiol..

[48] Faruque SM (1997). Molecular analysis of toxigenic Vibrio cholerae O139 Bengal strains isolated in Bangladesh between 1993 and 1996: evidence for emergence of a new clone of the Bengal vibrios. J. Clin. Microbiol..

[49] Senoh M (2014). Isolation of viable but nonculturable Vibrio cholerae O1 from environmental water samples in Kolkata, India, in a culturable state. MicrobiologyOpen.

[50] Zo Y-G (2002). Genomic profiles of clinical and environmental isolates of Vibrio cholerae O1 in cholera-endemic areas of Bangladesh. Proc. Natl. Acad. Sci. USA.

[51] Lessler, J. *et al*. Mapping the burden of cholera in sub-Saharan Africa and implications for control: an analysis of data across geographical scales. *The Lancet* **0** (2018). 10.1016/S0140-6736(17)33050-7 PMC5946088 29502905

